# Fragility Fractures: Risk Factors and Management in the Elderly

**DOI:** 10.3390/medicina57101119

**Published:** 2021-10-17

**Authors:** Filippo Migliorini, Riccardo Giorgino, Frank Hildebrand, Filippo Spiezia, Giuseppe Maria Peretti, Mario Alessandri-Bonetti, Jörg Eschweiler, Nicola Maffulli

**Affiliations:** 1 Department of Orthopaedics, Trauma and Reconstructive Surgery, RWTH Aachen University, 52074 Aachen, Germany; migliorini.md@gmail.com (F.M.); fhildebrand@ukaachen.de (F.H.); joeschweiler@ukaachen.de (J.E.); 2 Residency Program in Orthopedics and Traumatology, University of Milan, 20122 Milan, Italy; riccardo.giorgino@unimi.it; 3 Department of Orthopaedic and Trauma Surgery, Ospedale San Carlo Potenza, Via Potito Petrone, 85100 Potenza, Italy; spieziafilippo@gmail.com; 4 Department of Biomedical Sciences for Health, University of Milan, 20133 Milan, Italy; giuseppe.peretti@unimi.it; 5 IRCCS Orthopedic Institute Galeazzi, 20161 Milan, Italy; 6 Plastic, Reconstructive and Aesthetic Surgery, University of Milan, 20122 Milan, Italy; Mario.alessandri@unimi.it; 7 Department of Medicine, Surgery and Dentistry, University of Salerno, 84081 Baronissi, Italy; 8 School of Pharmacy and Bioengineering, Keele University Faculty of Medicine, Thornburrow Drive, Stoke-on-Trent ST4 7QB, UK; 9 Centre for Sports and Exercise Medicine, Barts and the London School of Medicine and Dentistry, Queen Mary University of London, Mile End Hospital, London E1 4DG, UK

**Keywords:** fragility, fractures, osteoporosis

## Abstract

Given the progressive ageing of Western populations, the fragility fractures market has a growing socioeconomic impact. Fragility fractures are common in the elderly, negatively impacting their quality of life, limiting autonomy, increasing disability, and decreasing life expectancy. Different causes contribute to the development of a fractures in frail individuals. Among all, targeting fragile patients before the development of a fracture may represent the greatest challenge, and current diagnostic tools suffer from limitations. This study summarizes the current evidence on the management of fragility fractures, discussing risk factors, prevention, diagnosis, and actual limitations of the clinical therapeutic options, putting forward new ideas for further scientific investigation.

## 1. Introduction

Fragility fractures are frequent in the elderly. The lifetime risk of osteoporotic fractures lies within the range of 40–50% in women and 13–22% for men [1,2], with mortality higher in men [3]. Patients aged 65 years and above suffer fragility status and increased risk of adverse health outcomes [4,5] such as impaired mobility, prolonged hospitalization, residual disability, and reduced life expectancy. Osteoporosis affects frail patient, increasing the risk of fracture for the remaining lifespan [6,7]. Fragility fractures also have a major economic impact. The more than 2 million osteoporosis-related fractures in 2005 in the United States, with 71% occurring in women and 29% in men, had a total cost of nearly $17 billion [8]. By 2025, fractures and associated costs are predicted to grow by more than 48% [8]. In Sweden, the total cost of fragility fractures is about 3.2% of total health-care cost [9]. The increasing number of elderly people in Western countries and the rising trend in terms of population aging is evident in clinical practice [7,8]. Consequently, osteoporotic fractures and frail patients are becoming an urgent challenge to healthcare providers [10].

This study summarizes current evidence on the management of fragility fractures, discussing risk factors, prevention, diagnosis, and actual limitations of the clinical therapeutic options, inspiring new ideas for further scientific investigation.

## 2. Fragility Fractures

Fragility fractures arise from low-energy trauma in daily activities, mostly occurring in the elderly [11,12]. In general, fragility fractures of the hip and spine have the worst impact on the health of the elderly [13,14,15]. However, many other areas of the body can also be affected by fragility fractures, such as humerus, pelvis, forearm, ribs, distal femur, tibia, and clavicle [16,17]. Quality of life is significantly impaired with a different impact depending on the location of the fracture. Hip fractures have devastating results, with a high 1-year mortality in both sexes [18,19,20], and a dramatic loss in personal independence [21,22]. Vertebral fractures, especially those affecting the thoraco-lumbar junction, are also responsible for worsening the quality of life [23,24,25], even though they may be asymptomatic [26]. While these former two sites of fractures have the worst consequences for the patient, other fracture sites are responsible for an increased risk of mortality [15,27].

## 3. Risk Factors

Different causes contribute to the development of fractures in frail individuals, and osteoporosis is a common underlying factor [28,29]. Osteoporosis impairs the Bone Mineral Density (BMD) with profound microarchitectural deterioration of bone that leads to increased fragility [30,31]. Bone densitometry or mineralometry is an imaging technique which makes it possible to assess and measure the density of bone using a very low dose of radiation [32]. To evaluate the BMD resulting from bone densitometry, the T and Z scores are used [32]. The T score compares the patient’s bone mineral density with the mean peak bone density of a 30-year-old person of the same sex [32]. The z score compares the patient’s bone mineral density with mean peak density for someone the same age [32]. According to the WHO, a spine, hip, or wrist BMD of 2.5 SD or more below the reference mean (T-score ≤ −2.5) is compatible with the diagnosis of osteoporosis [32]. In postmenopausal women, the increased fracture risk is highest after any clinical fracture, and the risk is independent of the location of the fracture [33]. Klotzbuecher et al. confirmed these results and showed that the risk of subsequent fracture is doubled [34]. Age is one of the most significant risk factors, responsible for an increased risk of fracture regardless of BMD [35], a consequence of bone aging but also of the comorbidities of elderly patients [36]. Gender also plays an important role in fracture risk: postmenopausal hormonal modification negatively impacts the quality of the bone [18]. In addition, females presented an increased risk of falling compared to males [37], itself an independent predictor of fragility fracture [38,39]. A further age-related factor that plays a fundamental role in fragility fractures lies in falls. Falls are extremely common in the frail population, but their likelihood is usually underestimated. Falls in the elderly dramatically impact life expectancy, as the associated complications are often severe, and the recovery process is prolonged and difficult to complete [40]. In addition to the parameters used to monitor the specific risk of falling, a previous fall also significantly impacts the patient’s functional decline [41].

## 4. Diagnostic Tools

Targeting fragile patients before the development of a fracture significantly impacts on their survival, and represents a great scientific challenge [42,43]. Usually, frail patients meet the doctor or are hospitalized for the first time when osteoporotic fractures occur, and consequently, when their life expectancy already dramatically diminished [20]. Indeed, initial patient assessment is usually performed with plain radiographs and later implemented with either CT scan or MRI, if necessary. While CT scan better characterizes the fracture in all its aspects (extension, 3D architecture, articular involvement), MRI gives more detailed information related to the timing of fracture onset, especially in vertebral fractures, according to the presence of oedema, spinal cord compression, and soft tissue involvement. Several tools are now available for the diagnosis of bone fragility, both clinically and radiographically. Dual-Energy X-ray Absorptiometry (DXA) remains the standard diagnostic tool to evaluate BMD through specific scores [44]. A low-BMD is an important predictor of fragility fractures [45,46]. However, some patients with fragility fractures do not have low levels of BMD [47]. In fact, fragility is not simply a reduction in bone quantity [48]. The Fracture Assessment Tool (FRAX^®^) is an online website free to use (https://www.sheffield.ac.uk/FRAX/index.aspx, accessed on September 2021). The FRAX^®^ is based on population-based cohort studies from Europe, North America, Asia, and Australia. The results demonstrate the 10-year probability of a hip fracture and of a major osteoporotic fracture (clinical spine, forearm, hip, or shoulder fracture) [49,50,51]. However, this software lacks to recognize the impact of other the risk of falling, such as loss of balance, reduced vision, and altered motor coordination. The great practical utility of FRAX is to indicate a risk threshold after which patients deserve medical intervention [52]. Furthermore, patient evaluation with Time Up and Go (TUG) and Tinetti tests allows to identify those patients at increased risk of falling because of their overall fragility, and it seems to correlate both with BMD and 10-year fracture risk. While the TUG test evaluates the time needed by the patient to get up from a chair, walk three meters, forwards and backwards, and to then sit back down, the Tinetti test considers both equilibrium and gait. A geriatric patient screening with the Identification of Seniors at Risk (ISAR) score can also be helpful in identifying frail patients and in predicting patient mortality. Recently, more and more studies suggest the importance of BTMs (bone turnover marker) in identifying unhealthy bone [53,54]. These markers help to evaluate the dynamics of bone resorption and formation in the osteoporotic bone. BTMs, such as bone alkaline phosphatase (bALP) and procollagen type I N propeptide (PINP), are used as biomarkers for bone ossification, while serum cross-linked C-telopeptides of type I collagen (bCTx) and urinary cross-linked N-telopeptides of type I collagen (NTx) for bone resorption [55,56]. Several endogenous factors, such as age, gender, diseases, ethnicity, and fractures exert an influence on the BTMs [57]. Exogenous factors, such as seasonal variation, exercise, circadian rhythm, and diet, influence BTMs [58,59]. Also, other than primary osteoporosis, other pathological conditions are responsible for fragility fractures, such as endocrine disorders, collagen disorders, vitamin deficiencies, drugs, and hormonal therapies [60,61,62,63,64,65].

## 5. Treatments

### 5.1. Ortho-Geriatric Comanagement

The management of fragility fractures should be focused on the individual frail patient [66]. A fragility fracture in the elderly represents a significant problem precisely in relation to the fragile nature of the patient, a subject with numerous comorbidities who would need to be treated in the entirety of their critical clinical picture by a specialized team. Some studies showed the primacy of the interdisciplinary approach to these patients so that trauma surgeons should work in synergy with other professionals such as nurses, physiotherapists, occupational therapists, and social services [67,68,69]. Other recent studies analyzed the activity of certified Geriatric Trauma Center in accordance with the guidelines of the German Trauma Society (DGU), where the patient is treated by a multidisciplinary team, showing clear advantages in patients receiving multidisciplinary orthogeriatric treatment, especially in terms of lower rates of cardiorespiratory complications and mortality [70,71,72]. The so-called ortho-geriatric comanagement is central in the treatment process of these kind of patients. This favorably affects outcomes and economics compared to that of the standard treatment of a single discipline, especially in proximal femoral fractures. Indeed, shorter length of stay, fewer complications and readmission rates, and higher patient satisfaction were witnessed when this approach was used. In fact, other conditions can be associated with fragility fractures, such as peripheral arteriopathy, present in about 20% of geriatric patients [73]. In case of surgery for fragility fractures on the lower limbs, measures to improve peripheral circulation should be considered to reduce soft tissue complications [74].

### 5.2. Timely Surgery

Most fragility fractures have to be managed surgically, especially when the lower extremity is involved. In most cases, patients should be operated within 24 hours, and no later than 48 hours, after admission [75,76]. It is, therefore, essential to optimize patient management times and coordinate pre- and postoperative guidelines. After surgical stabilization, most patients achieve higher level of function and freedom from pain compared to that of conservative management [77]. On the other hand, when considering upper limb fragility fractures, mainly proximal humerus or distal radius, nonoperative treatment remains the gold standard, especially when the fracture is not displaced. The rationale behind this difference is that, while weight-bearing is of uttermost importance in the quality of life of an individual and can only be achieved through surgical management, the upper limb range of motion is not considerably altered when operative and nonoperative treatments are compared.

### 5.3. Full Weight-Bearing

In the frail population, surgical management, especially in lower limb fractures, should aim to restore the activity level of the individual and maximize weight-bearing ability rather than to achieve the best fracture reduction. Thus, sometimes it is better to completely replace the joint with a prosthesis rather than using an open reduction internal fixation (ORIF) technique, which would require longer rehabilitation [78,79,80]. When ORIF is used, supplemental fixation with longer plates and more screws and cables is warranted.

### 5.4. Specific Implants and Augmentation

Regarding the surgical aspect, the typical site of surgical failure of fragility fractures is at the interface between bone and implant. This occurs because of decreased BMD, cortical bone thinning, increased incidence of shredding despite the low-energy injury mechanism, and increased healing time in osteoporotic bone [81,82,83], which is more brittle and less elastic. For these reasons, the literature suggests the use of specific implants, such angle stable implants, which provide higher stability [83,84,85]. Other studies underlined the importance of bone augmentation to increase the overall stability of the structure and improve implant fixation [86]. Bone grafting, either biologic or synthetic, bone cements, or hydroxyapatite coatings are often needed. Tricalcium phosphate and polymethylmethacrylate (PMMA) were used to increase contact area and strengthen the anchorage of the implant. In any case, complications may arise, such as lack of integration or the possible development of heat during curing, which is potentially harmful to the surrounding soft tissues, and the risk of thermal bone necrosis [87].

### 5.5. Early Rehabilitation

Even though most patients cannot simply be discharged immediately after the fracture is operated on, rehabilitation facilities are not always able to cope with the mass of patients. Therefore, hospital management of these fractures should aim to increase mobility and active skills in daily life to quickly discharge patients directly to home. Indeed, the goal in the management of these fracture is to allow a fast recovery, diminish disability, and avoid patient immobilization [88,89,90].

### 5.6. Fall Prevention/Preventive Surgery

In addition to mortality risk, a fragility fracture is a major risk factor for further fractures [85,86]. Therefore, further interventions to not only hinder osteoporosis progression but also to prevent falls are necessary [91,92]. Pharmacological interventions targeting osteoporosis include calcium and vitamin D supplementation, bisphosphonates, PTH agonists, and the RANKL inhibitor [49,91]. Considering fall prevention, continued patient screening with TUG test and follow-up, home hazard reduction, implementation of exercise programs, and eventually, preventive surgery could lower the risk of further fractures. Among preventive surgery techniques, femoroplasty is the most studied. It involves the injection of bone cement in the proximal femur, which seems to increase load to fracture and to correlate to the amount of cement filling.

As mentioned, falls represent a most important risk factors and dramatically impact life expectancy [40,41]. Thus, fall risk assessment is of uttermost importance as a preventive measure and for better patient evaluation to lower not only fragility fracture incidence, but also morbidity and mortality. Being able to reduce falls as much as possible is a major challenge for the health system, and preventive measures are necessary to educate individuals at risk. Therefore, in addition to rapid fall risk assessment tools such as the Aachen Falls Prevention Scale (AFPS) [93], new assessment scales were proposed, such as the Aachen Mobility and Balance Index (AMBI), which includes mobility and difficulty tasks and a measurement of the grip strength of the dominant hand [94]. Furthermore, promising results were found in smartphone apps for fall prevention [95].

## 6. Conclusions

Fragility fractures are a widespread condition of fundamental importance in the global health system. Fragility fractures are common, and appropriate management minimizes their morbidity health expenditures. Evidence from forecasting models also highlights the possibility of increasing life expectancy, and the need for careful planning for health and social services is urgent. Age is one of the most important risk factors for the development of fragility fractures. Prevention is the key to master their management, including the use of drugs against osteoporosis. Treatment indications should be available to general practitioners together with improvement to standardize timing of surgery. An interdisciplinary approach improves the outcomes of frail patients. The early diagnosis of patients at risk to prevent future fractures in the frail population represents a pivotal point in prevention, and this can be achieved by improving the communication between general practitioners and orthopedic surgeons.

## Data Availability

The data presented in this study are available within the manusctipt.
